# Diagnosis of Sarcopenia Using Convolutional Neural Network Models Based on Muscle Ultrasound Images: Prospective Multicenter Study

**DOI:** 10.2196/70545

**Published:** 2025-05-06

**Authors:** Zi-Tong Chen, Xiao-Long Li, Feng-Shan Jin, Yi-Lei Shi, Lei Zhang, Hao-Hao Yin, Yu-Li Zhu, Xin-Yi Tang, Xi-Yuan Lin, Bei-Lei Lu, Qun Wang, Li-Ping Sun, Xiao-Xiang Zhu, Li Qiu, Hui-Xiong Xu, Le-Hang Guo

**Affiliations:** 1 Department of Ultrasound, Zhongshan Hospital Institute of Ultrasound in Medicine and Engineering Fudan University Shanghai China; 2 Department of Medical Ultrasound Shanghai Tenth People’s Hospital Shanghai China; 3 Ultrasound Research and Education Institute Tongji University School of Medicine Shanghai China; 4 Shanghai Engineering Research Center of Ultrasound Diagnosis and Treatment Shanghai China; 5 MedAI Technology (Wuxi) Co, Ltd Wuxi China; 6 Department of Medical Ultrasound West China Hospital of Sichuan University Chengdu China; 7 Chair of Data Science in Earth Observation Technical University of Munich Munich Germany

**Keywords:** ultrasound, sarcopenia, artificial intelligence, convolutional neural network, multicenter study

## Abstract

**Background:**

Early detection is clinically crucial for the strategic handling of sarcopenia, yet the screening process, which includes assessments of muscle mass, strength, and function, remains complex and difficult to access.

**Objective:**

This study aims to develop a convolutional neural network model based on ultrasound images to simplify the diagnostic process and promote its accessibility.

**Methods:**

This study prospectively evaluated 357 participants (101 with sarcopenia and 256 without sarcopenia) for training, encompassing three types of data: muscle ultrasound images, clinical information, and laboratory information. Three monomodal models based on each data type were developed in the training cohort. The data type with the best diagnostic performance was selected to develop the bimodal and multimodal model by adding another one or two data types. Subsequently, the diagnostic performance of the above models was compared. The contribution ratios of different data types were further analyzed for the multimodal model. A sensitivity analysis was performed by excluding 86 cases with missing values and retaining 271 complete cases for robustness validation. By comprehensive comparison, we finally identified the optimal model (SARCO model) as the convenient solution. Moreover, the SARCO model underwent an external validation with 145 participants (68 with sarcopenia and 77 without sarcopenia) and a proof-of-concept validation with 82 participants (19 with sarcopenia and 63 without sarcopenia) from two other hospitals.

**Results:**

The monomodal model based on ultrasound images achieved the highest area under the receiver operator characteristic curve (AUC) of 0.827 and F1-score of 0.738 among the three monomodal models. Sensitivity analysis on complete data further confirmed the superiority of the ultrasound images model (AUC: 0.851; F1-score: 0.698). The performance of the multimodal model demonstrated statistical differences compared to the best monomodal model (AUC: 0.845 vs 0.827; P=.02) as well as the two bimodal models based on ultrasound images+clinical information (AUC: 0.845 vs 0.826; P=.03) and ultrasound images+laboratory information (AUC: 0.845 vs 0.832, P=0.035). On the other hand, ultrasound images contributed the most evidence for diagnosing sarcopenia (0.787) and nonsarcopenia (0.823) in the multimodal models. Sensitivity analysis showed consistent performance trends, with ultrasound images remaining the dominant contributor (Shapley additive explanation values: 0.810 for sarcopenia and 0.795 for nonsarcopenia). After comprehensive clinical analysis, the monomodal model based on ultrasound images was identified as the SARCO model. Subsequently, the SARCO model achieved satisfactory prediction performance in the external validation and proof-of-concept validation, with AUCs of 0.801 and 0.757 and F1-scores of 0.727 and 0.666, respectively.

**Conclusions:**

All three types of data contributed to sarcopenia diagnosis, while ultrasound images played a dominant role in model decision-making. The SARCO model based on ultrasound images is potentially the most convenient solution for diagnosing sarcopenia.

**Trial Registration:**

Chinese Clinical Trial Registry ChiCTR2300073651; https://www.chictr.org.cn/showproj.html?proj=199199

## Introduction

Sarcopenia is characterized by significant loss of skeletal muscle mass with decreased strength and functional performance [1]. It is associated with worse clinical outcomes, such as elevated mortality rates, increased falls, higher incidences of hospitalization, and shorter overall survival of cancers [2,3]. It is considered one of the leading contributors to depression, social isolation, frailty, and invalidity, which imposes a growing burden on public health and social services [4]. The clinical diagnosis involves multiple intricate steps, such as dual-energy x-ray absorptiometry, handgrip strength, 5-time chair stand test, and more [1]. Consequently, it is challenging for older individuals, especially those who are bedridden or receiving home health care, to complete the entire diagnostic process in actual clinical practice.

Sarcopenia significantly influences the secretion and metabolism of the body, such as glucose, glycogen, and lipid metabolism, as well as the secretion of a variety of cytokines [5]. Therefore, clinical data from laboratory information may provide supplementary information for the diagnosis of sarcopenia. Besides the metabolic changes, the pathogenesis of sarcopenia primarily encompasses fiber loss, atrophy, and increased infiltration of adipose and connective tissue [6,7]. Therefore, microalterations in sarcopenia may cause macroscopic changes in muscle characteristics, like fiber mass and morphology, which facilitate the detection of sarcopenia by imaging.

Ultrasound is increasingly being used for muscle assessment [8,9], not only because of its portability, cost-effectiveness, and real-time performance but also because ultrasound images’ texture and volume change provide detailed visualization information of the abovementioned macroscopic changes in the muscle. However, the qualitative or quantitative assessment of muscle image texture, grayscale, and volume change is highly subjective and time-consuming for humans [10-12]. To effectively diagnose and manage sarcopenia, there is an urgent need to develop an objective, simple, and accurate method for identifying the characteristic changes of sarcopenia on ultrasound images.

Convolutional neural networks (CNNs) are increasingly preferred due to their outstanding performance and high reproducibility in medical image analysis tasks [13]. Similarly, the objective, hard-to-quantify, and minor changes in ultrasound images on texture and volume in muscle may be quickly captured by CNN methods. Some CNN models using ultrasound images have exhibited remarkable capabilities in muscle segmentation, muscle function assessment, and myositis diagnosis [14-16]. However, the potential of CNN models based on ultrasound images for sarcopenia diagnosis remains underexplored and insufficiently evaluated.

To conveniently and effectively screen for sarcopenia, we hypothesize that CNN models incorporating ultrasound images, clinical information, and laboratory information can achieve reliable diagnostic performance. In this study, we developed and validated several CNN models with different modalities, comparing their effectiveness through internal validation, external validation, and proof-of-concept analysis.

## Methods

### Ethical Considerations

This study was approved by the institutional ethics committees of the participating centers (Shanghai Tenth People’s Hospital, West China Hospital of Sichuan University, Zhongshan Hospital of Fudan University; SHSY-IEC-5.0/23K56/P01). All participants provided written informed consent before enrollment. Participants were informed of the study’s purpose, procedures, potential risks, and benefits. Privacy and confidentiality were strictly protected throughout the study; all data were anonymized before analysis and stored securely. No monetary or material compensation was provided to participants for their involvement in the study. The research protocol was registered at the Chinese Clinical Trial Registry (ChiCTR2300073651).

### Study Design

This was a prospective multicenter study. This study was designed and reported in accordance with the CONSORT-EHEALTH (Consolidated Standards of Reporting Trials of Electronic and Mobile Health Applications and Online Telehealth) guidelines [17]. In this study, we developed three monomodal models, two bimodal models, and one multimodal model based on ultrasound images, clinical information, and laboratory information.

**Figure 1:**
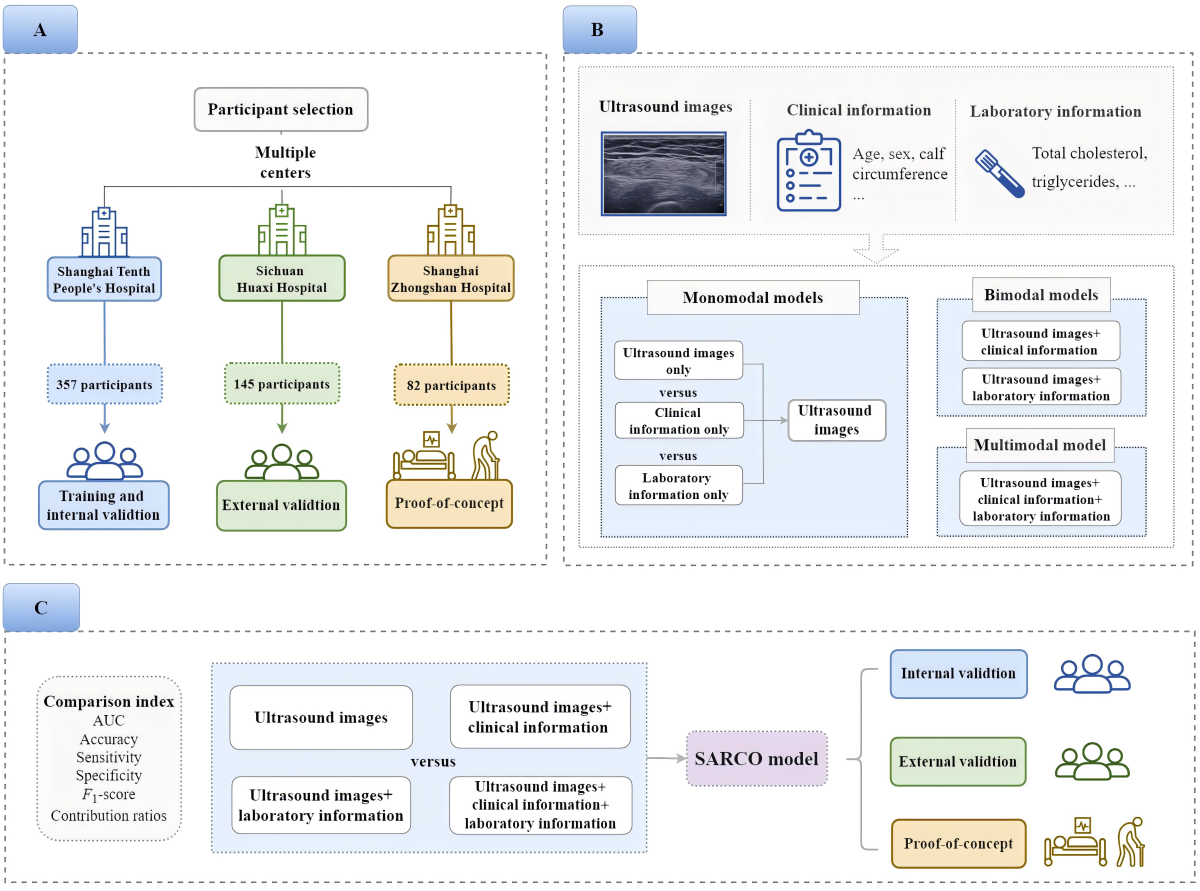
Overview of the study design. (A) The population characteristics of the different cohorts. In the training and internal validation cohort and the external validation cohort, participants were suspected to have sarcopenia and able to complete muscle strength and physical performance assessments. In the proof-of-concept cohort, participants were weak, bedridden, or had other bodily dysfunction and unable to complete muscle strength and physical performance assessments. (B) The candidate monomodal, bimodal, and multimodal models for the SARCO model. (C) The SARCO model was selected by comparing the diagnostic performance of the candidate monomodal, bimodal, and multimodal models, and the SARCO model was evaluated with external validation and proof-of-concept cohorts. AUC: area under the receiver operator characteristic curve.

First, three candidate monomodal models were developed and evaluated. The monomodal model with the best performance was selected as the basis. Second, we combined the best monomodal model with one of the other two data types to develop two candidate bimodal models. Finally, we combined the three data types to develop one multimodal model. After 5-fold cross-internal validation, the diagnostic performance of the candidate monomodal, bimodal, and multimodal models were compared. Moreover, the relative contributions of each data type to the overall classification results were also analyzed. Finally, the optimal candidate model was determined as the SARCO model by comprehensive assessment. The SARCO model was performed with external validation and proof-of-concept validation. Figure 1 illustrates a comprehensive design of the study.

### Study Population

The population characteristics of the different cohorts are shown in Figure 1A. From October 2021 to December 2022, a total of 2735 ultrasound images and the associated clinical data from 357 participants were collected for training and internal validation in Shanghai Tenth People’s Hospital. From May 2023 to December 2023, a total of 352 ultrasound images from 145 participants were prospectively collected for external validation of the model in the West China Hospital, Sichuan University. The inclusion criteria were as follows: (1) aged 60 years or older and (2) participants with suspected sarcopenia, for example, functional decline or limitation, unintentional weight loss, and diabetes mellitus [1]. The exclusion criteria were as follows: (1) inability to complete muscle strength and physical performance assessments and (2) not agreeing to participate in this study.

From October 2023 to January 2024, a total of 817 ultrasound images from 82 participants were prospectively collected for proof-of-concept validation of the model in Zhongshan Hospital, Fudan University. The inclusion criteria were as follows: (1) aged 60 years and older; (2) participants with decompensated cirrhosis [18]; (3) inability to complete muscle strength and physical performance assessments due to weakness, bedridden, or other bodily dysfunction; and (4) available abdominal computed tomography (CT) scan from which an adequate image at the L3 level could be obtained. The exclusion criteria were as follows: (1) without abdominal CT scan or poor image quality and (2) not agreeing to participate in this study. The flow diagram of the inclusion and exclusion is shown in Multimedia Appendix 1.

### Sarcopenia Assessment

The assessment of participants in Shanghai Tenth People’s Hospital and West China Hospital was based on the consensus of the Asian Working Group for Sarcopenia, where the reference criteria for the diagnosis of sarcopenia include low muscle mass, low muscle strength, and/or low physical performance [1]. Extremity muscle mass was determined using the logic Discovery Wi dual-energy x-ray bone densitometer (Hologic Discovery QDR Series) or the Inbody 770 body composition analyzer. The Appendicular Skeletal Muscle Mass Index was calculated using the following formula:

Appendicular Skeletal Muscle Mass Index=extremity muscle mass (kg)/height squared (m^2^)

Handgrip strength (kg) was measured by the Camry electronic grip strength meter (EH101; Camry Scale). Physical performance was assessed by 6-meter walking speed (m/s) and 5-time chair stand test (s). The participants at Zhongshan Hospital were assessed for sarcopenia using the L3-skeletal muscle index by CT. The cutoff values for diagnosing sarcopenia were 44.77 cm^2^/m^2^ for men and 32.50 cm^2^/m^2^ for women [18].

### Clinical Information and Laboratory Information

The clinical information included the presence of diabetes, the presence of osteoporosis, age (years), gender, height (m), weight (kg), BMI (kg/m^2^), and calf circumference (cm). The laboratory information was obtained before muscle strength and physical function assessments. The laboratory information included total cholesterol (mmol/L); triglycerides (mmol/L); high-density lipoprotein (mmol/L); low-density lipoprotein (mmol/L); glycosylated hemoglobin (%); albumin (g/L); hemoglobin (g/L); 1,25(OH)2D (ng/mL); osteocalcin (ng/mL); free triiodothyronine (pmol/L); free tetraiodothyronine (pmol/L); total triiodothyronine (nmol/L); total tetraiodothyronine (nmol/L); and thyroid-stimulating hormone (mIU/L).

### Ultrasound Examination

The ultrasound equipment and transducer of the centers are shown in Multimedia Appendix 2. The ultrasound equipment in the training and internal validation cohort was the Aixplorer Ultrasound system (SuperSonic Imagine), with an SL 10-2 multifrequency linear transducer. In the external validation cohort, the ultrasound equipment was the Aixplorer Ultrasound system (SuperSonic Imagine) with an SL 10-2 multifrequency linear transducer, the Mindray M9 Portable Ultrasound Machine (Mindray) with an SL 10-3 multifrequency linear transducer, and the Mindray MX7 Portable Ultrasound Machine (Mindray) with an SL 13-3 multifrequency linear transducer. In the proof-of-concept cohort, the ultrasound equipment was the R10 Prestige ultrasound system (Samsung Medison Co. Ltd) with an SL 12-3 multifrequency linear transducer. All participants underwent ultrasound examination with radiologists with 5 years or more of experience in musculoskeletal ultrasound. The radiologists reached a consensus on the examination procedure.

The participants were placed in the supine position. The rectus femoris (RF) was chosen for ultrasound examination, with the transducer positioned at the middle position between the anterior superior iliac spine and the lower edge of the patella. The musculoskeletal mode was preset, and the gain, depth, and focus were adjusted to visualize the muscle properly. The transducer was oriented perpendicularly to the long axis of the RF to capture three to five cross-sectional images of RF. The ultrasound images were saved in DICOM or JPG format for further processing and analysis.

### Data Preprocess

#### Image Data Processing

In order to reduce the influence of other tissues on the feature extraction of the deep learning network, a single and experienced radiologist manually delineates the muscle areas on all images for separation with the ITK-SNAP software (version 3.8.0; Penn Image Computing and Science Laboratory, University of Pennsylvania). The region of interest was defined based on smooth and clear anatomical landmarks of muscle fascia to ensure accurate segmentation of the muscle regions. The muscle segmentation process and regions on ultrasound images are shown in the Multimedia Appendix 3. To ensure consistent dimensions for CNN models, all ultrasound images were resized to 224×224 pixels. Before input into the CNN models, the pixel values of the ultrasound images were normalized to fall within the range of 0 to 1. We used web-based data augmentation methods on the ultrasound images to expand the dataset and mitigate overfitting. These methods included random horizontal or vertical flipping, random shifting (shift limit of 0.1), random scaling (scale limit of 0.1), and random rotation (–10° to 10°). In addition, random adjustments to brightness and random contrast were also applied to improve the generalization of CNN models.

#### Clinical Information and Laboratory Information Processing

For each feature, missing data were imputed with a mean value. Outliers were detected using the *z* score statistical method and subsequently corrected manually. Discrete characteristic variables, such as gender, were encoded using one-hot encoding, while continuous characteristic variables were normalized to a range between 0 and 1. After preprocessing, the clinical data were represented as a 22D feature vector.

### Model Development and Architecture

#### Development and Architecture of CNN Models Based on Ultrasound Images

**Figure 2:**
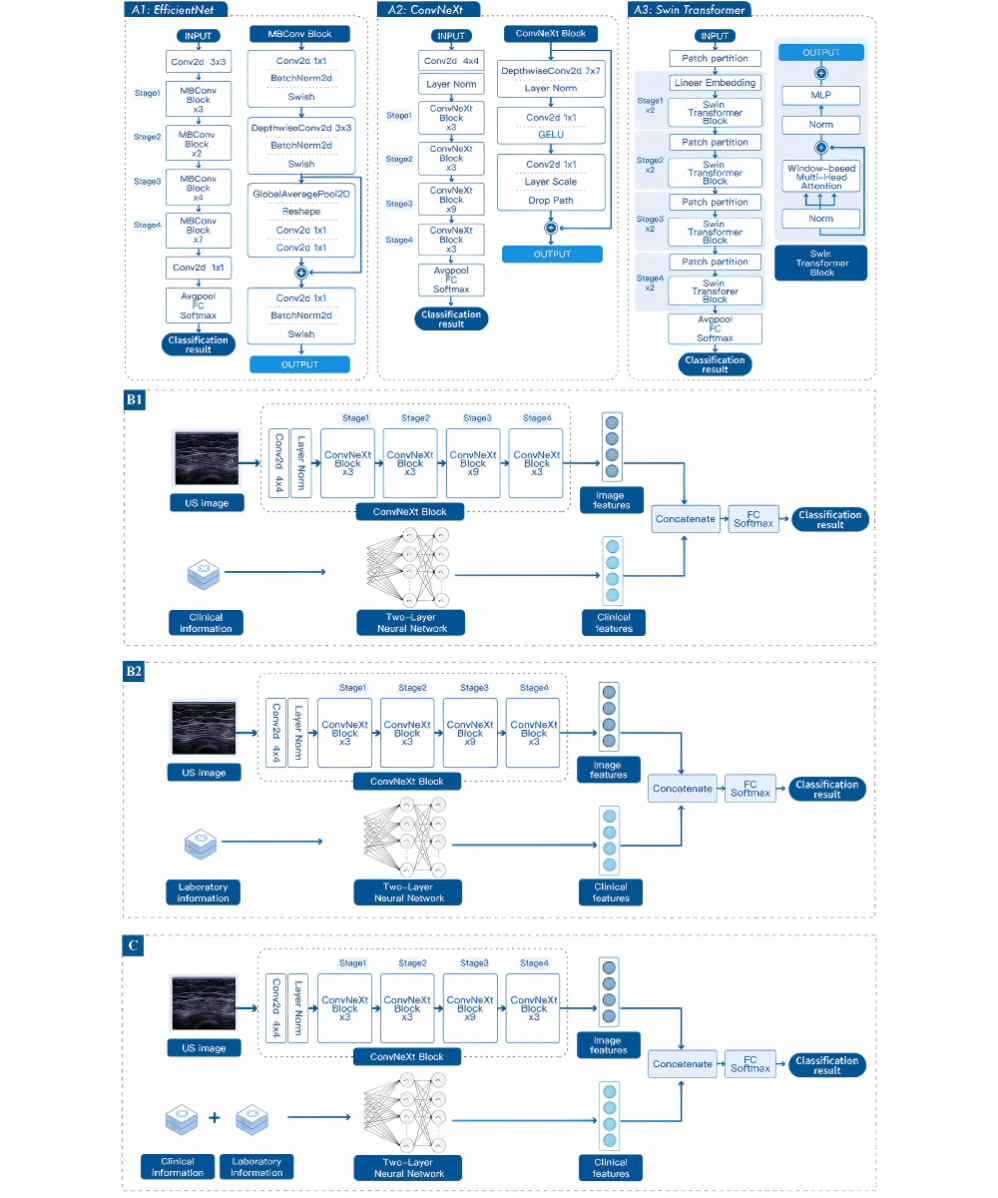
The development and architecture of three monomodal models for the ultrasound images and the development and architecture of two bimodal models and one multimodal model. (A1) The development and architecture of the EfficientNet. (A2) The development and architecture of the ConvNeXt. (A3) The development and architecture of the Swin Transformer. (B1) The development and architecture of the bimodal model of the ultrasound images and clinical information. (B2) The development and architecture of the bimodal model of the ultrasound images and laboratory information. (C) The development and architecture of the multimodal model of the ultrasound images, clinical information, and laboratory information.

Three state-of-the-art image classification models (EfficientNet, ConvNeXt, and Swin Transformer) were used for ultrasound image feature extraction and sarcopenia classification. The details of the developments of the three models are shown in Multimedia Appendix 4. The developments and architecture of the three models are shown in Figure 2A. The pretrained weights of all models were obtained from training a subset of the ImageNet dataset. The final fully connected and softmax layer was replaced with a new fully connected layer, and the pretrained models were fine-tuned with the ultrasound training dataset.

#### Development and Architecture of Clinical Information Multilayer Perceptron Model

The multilayer perceptron (MLP) network for clinical information is designed with an input layer corresponding to the 8D clinical feature vector. A hidden layer with a dimensionality of 128, using the Gaussian error linear unit (GELU) activation function, is used. GELU smoothly weights inputs by their probability under a Gaussian distribution, leading to improved training stability and performance in deep learning models. Additionally, a dropout layer is added for regularization. The output layer is a binary classifier indicating the presence or absence of muscular dystrophy.

#### Development and Architecture of Laboratory Information MLP Model

The MLP network for laboratory information is designed with an input layer corresponding to the 14D laboratory feature vector. Similar to the clinical model, a hidden layer with a dimensionality of 128, GELU activation function, and dropout layer are used. The output layer serves as a binary classifier.

#### Development and Architecture of Bimodal and Multimodal CNN Models

The two bimodal models are illustrated in Figure 2B, and the multimodal model is illustrated in Figure 2C. Both the bimodal and multimodal models comprise two branches. Ultrasound images were first passed through the pretrained network to extract image features. Clinical and laboratory information is passed through a simple two-layer, feed-forward neural network to capture essential features and concatenate them with the extracted image-based features. Finally, the concatenation was passed through an output layer with a sigmoid activation to estimate the probability of each diagnosis.

### Implementation

All experiments were conducted using Python 3.8.3 and Pytorch 1.7.0 on a 64-bit Ubuntu 18.04.4 LTS operating system. We used two NVIDIA TITAN graphics processing units with 24 GB of memory for training. The batch size used was 48, with a 3×10^–4^ learning rate. We used a warm-up strategy at the beginning of the training and the CosineAnnealingLR learning rate decay strategy. The maximum number of epochs was set as 150. AdamW was used as an optimizer, and the loss function we chose is binary cross-entropy.

### Training and Validation of CNN Models

In order to minimize experimental errors arising from discrepancies in data distribution, we adopted a 5-fold cross-validation approach to evaluate the performance of CNN models. The data were partitioned at the participant level into five subsets. Subsequently, four subsets were designated as the training set, with the remaining one as the validation set. Recognizing the imbalance between positive and negative images, we introduced random down-sampling of negative images within the training data, ensuring parity with the number of positive images. This balanced dataset was then used to train the model. Finally, we applied a model ensemble technique to merge these three sets of trained weights, creating an integrated model. This integrated model was then used to evaluate performance on the validation set.

### Heat Map Generation

To better interpret the CNN prediction results, the gradient-weighted class activation mapping method was used to generate heat maps. Heat maps can visualize the image’s most indicative areas to interpret the CNN models’ predictive mechanism, which reflects the contribution of each pixel in the ultrasound images to the prediction of sarcopenias. All heat maps were produced by applying the packages *Matplotlib* v3.8.0 and *TorchCAM* v0.3.2.

### Statistical Analysis

All statistical analyses were conducted using Python (version 3.6.13; Python Software Foundation), sci-kit-learn (version 0.20.0), and SPSS (version 26.0; developed by IBM Corp). Data were presented as mean (SD), median (IQR), or number (%) as appropriate. Comparisons of differences in clinical factors between groups with or without sarcopenia were analyzed using the independent 2-sample *t* test, Wilcoxon rank-sum test, or chi-square test. The receiver operating characteristic (ROC) curves were constructed to assess the prediction performance of models. The area under the ROC curve (AUC) with 95% CIs, sensitivity, specificity, and accuracy were investigated. The *F*_1_-score was also calculated:

F1-score= 2(precision×recall)/(precision+recall)

The Delong test was performed to compare the AUCs. Statistical significance was set at 2-sided *P*<.05. To comprehensively assess the impact of multimodal data on classification outcomes, we used the PyTorch Shapley additive explanations (SHAP) library for a detailed statistical analysis. Following model training, SHAP values were computed using the PyTorch SHAP library. The SHAP values were analyzed to understand the relative contributions of each modality to the overall classification results. By comparing the magnitudes of SHAP values across modalities, we quantified the influence of ultrasound images, clinical information, and laboratory data on the model’s decision-making process.

## Results

### Patient Characteristics

We enrolled a total of 357 participants (median age 67.00, IQR, 63.00-72.00 years), including 101 participants with sarcopenia (608 ultrasound images) and 256 participants without sarcopenia (2127 ultrasound images), in the training and internal validation cohort. In the external validation cohort, we enrolled a total of 145 participants (median age 71.00, IQR 67.00-77.00 years), including 68 participants with sarcopenia (70 ultrasound images) and 77 participants without sarcopenia (252 ultrasound images). In the proof-of-concept group, we enrolled a total of 82 participants (median age 66.00, IQR 63.00-71.00 years), including 19 participants with sarcopenia (185 ultrasound images) and 63 participants without sarcopenia (632 ultrasound images).

The clinical and laboratory information of the training and internal validation cohort is shown in Table 1. The age of participants with sarcopenia was older than that of those without sarcopenia (median age 72.00, IQR 66.00-80.50 vs 66.00, IQR 62.00-69.75 years; *P*<.001). Participants with sarcopenia had lower BMI, reduced calf circumference, and a lower prevalence of diabetes (all *P*<.05). Regarding laboratory information, patients with sarcopenia demonstrated lower levels of albumin, hemoglobin, and free triiodothyronine but higher levels of thyroid-stimulating hormone (all *P*<.05).

**Table 1:** The clinical and laboratory information of training and internal validation cohort.

				Without sarcopenia (n=256)		Sarcopenia (n=101)		*P* value	
**Clinical information**									
	Age (years), median (IQR)		66.00 (62.00-69.75)		72.00 (66.00-80.50)		<.001		
	**Sex, n (%)**								.30
		Male	117 (45.7%)		53 (52.5%)				
		Female	139 (54.3%)		48 (47.5%)				
	Height (m), median (IQR)		1.64 (1.58-1.70)		1.63 (1.57-1.70)		.43		
	Weight (kg), median (IQR)		66.75 (57.85-75.30)		62.50 (55.45-70.25)		.006		
	BMI (kg/m^2^), median (IQR)		24.70 (22.48-26.89)		23.53 (21.45-25.25)		.002		
	Calf circumference (cm), median (IQR)		35.00 (22.48-26.90)		33.34 (32.90-34.00)		<.001		
	ASMI^a^ (kg/m^2^), median (IQR)		5.40 (4.74-6.01)		4.84 (4.60-5.10)		<.001		
	Handgrip strength (kg), median (IQR)		29.00 (22.93-36.55)		20.84 (17.25-23.30)		<.001		
	6-meter walking speed (m/s), median (IQR)		1.16 (0.99-1.26)		1.03 (0.95-1.08)		<.001		
	5-time chair stand test (s), median (IQR)		9.38 (8.32-11.02)		12.76 (11.36-13.20)		<.001		
	Diabetes, n (%)		208 (81.3%)		72 (71.3%)		.04		
	Osteoporosis, n (%)		79 (30.9%)		41 (40.6%)		.08		
**Laboratory information, median (IQR)**									
	TC^b^ (mmol/L)		4.31 (3.72-5.01)		4.39 (3.57-4.95)		.90		
	TG^c^ (mmol/L)		1.32 (0.96-2.08)		1.28 (0.91-1.77)		.12		
	HDL^d^ (mmol/L)		1.17 (0.99-1.40)		1.20 (1.00-1.50)		.15		
	LDL^e^ (mmol/L)		2.50 (2.00-3.06)		2.60 (1.90-3.12)		.75		
	GHb^f^ (%)		8.28 (6.69-9.91)		7.98 (6.31-9.64)		.22		
	ALB^g^ (g/L)		39.90 (38.00-41.98)		37.60 (35.55-39.75)		<.001		
	HB^h^ (g/L)		132.00 (122.00-145.00)		124.00 (115.00-133.50)		<.001		
	1,25(OH)2D (ng/mL)		22.06 (17.06-33.50)		21.10 (27.37-29.63)		.05		
	OC^i^ (ng/mL)		11.75 (9.73-14.87)		12.12 (9.89-12.61)		.67		
	FT3^j^ (pmol/L)		4.84 (4.44-5.16)		4.64 (4.42-4.97)		.01		
	FT4^k^ (pmol/L)		15.27 (13.90-16.65)		14.99 (13.65-16.35）		.54		
	TT3^l^ (nmol/L)		1.51 (1.32-1.70)		1.51 (1.32-1.59)		.19		
	TT4^m^ (nmol/L)		97.55 (86.85-109.55)		99.70 (90.20-111.35)		.20		
	TSH^n^ (mIU/L)		1.69 (1.19-2.59)		2.03 (1.40-3.02)		.03		

^a^ASMI: appendicular skeletal muscle index.

^b^TC: total cholesterol.

^c^TG: triglycerides.

^d^HDL: high-density lipoprotein.

^e^LDL: low-density lipoprotein.

^f^GHb: glycosylated hemoglobin.

^g^ALB: albumin.

^h^HB: hemoglobin.

^i^OC: osteocalcin.

^j^FT3: free triiodothyronine.

^k^FT4: free tetraiodothyronine.

^l^TT3: total triiodothyronine.

^m^TT4: total tetraiodothyronine.

^n^TSH: thyroid stimulating hormone.

### The Process of Building and Selecting the Optimal Model

#### Selecting the Best Candidate Monomodal Model

The diagnostic performance of the 5-fold cross-validation of three networks based on ultrasound images is shown in Multimedia Appendix 5. The comparison of the AUCs and the *F*_1_-score of three networks based on ultrasound images are presented in Multimedia Appendix 6. The ConvNeXt and the Swim Transformer showed the top two diagnostic performances (AUC: 0.827 vs 0.835; *P*=.10); however, the ConvNeXt achieved the highest *F*_1_-score of 0.738 of the three networks. Then, the diagnostic performance of the ConvNeXt monomodal model based on ultrasound images was compared with that of the monomodal models with the other two data types.

**Figure 3:**
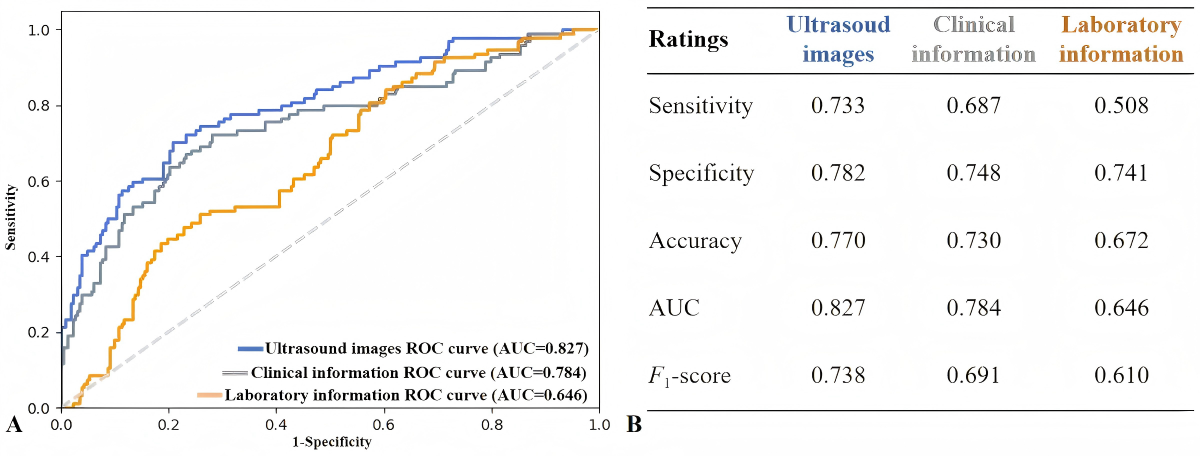
The diagnostic performance of three monomodal models. (A) The ROC curves of three monomodal models based on ultrasound images, clinical information, and laboratory information. (B) The diagnostic performance of three monomodal models based on ultrasound images, clinical information, and laboratory information. AUC: area under the receiver operator characteristic curve; ROC: receiver operating characteristic.

The diagnostic performance of the mean of the 5-fold cross-validation of the three monomodal models is shown in Figure 3. The clinical information model achieved an AUC of 0.784 and an *F*_1_-score of 0.691; the laboratory information model achieved an AUC of 0.646 and an *F*_1_-score of 0.610. The ultrasound images model achieved the highest AUC of 0.827 and *F*_1_-score of 0.738, which is regarded as the basis of the bimodal and multimodal models.

#### Comparison of the Monomodal, Bimodal, and Multimodal CNN Models

**Figure 4:**
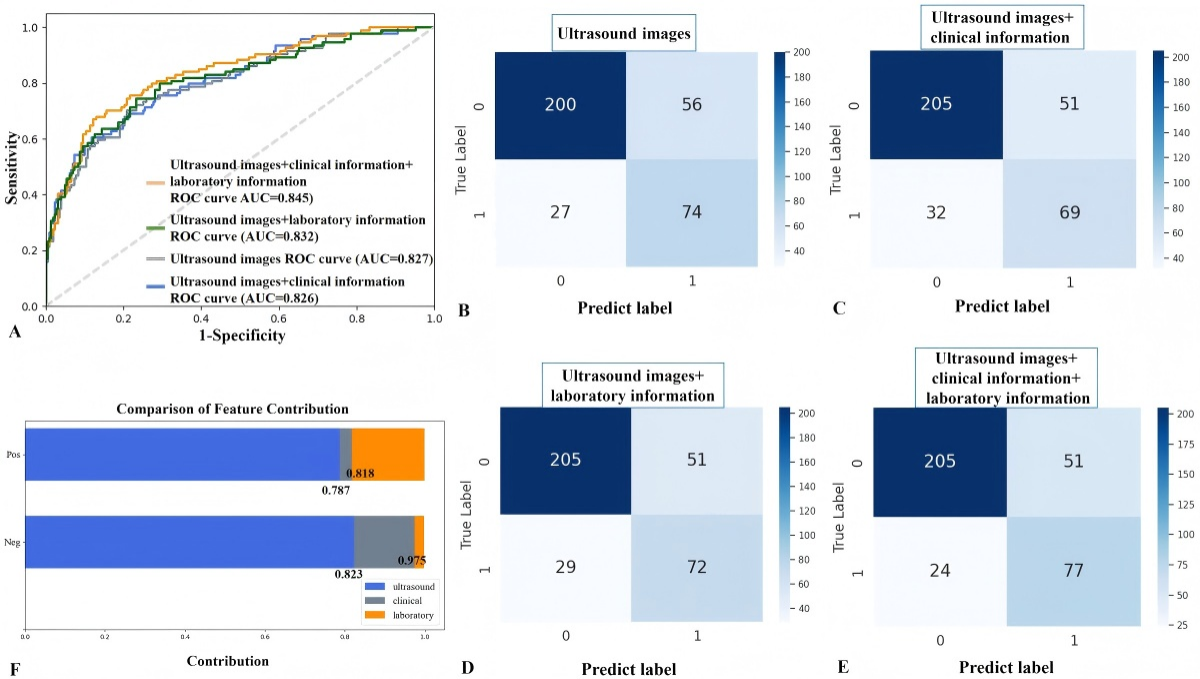
The diagnostic performance of the monomodal, bimodal, and multimodal models. (A) The ROC curves of the monomodal, bimodal, and multimodal models. (B) The confusion matrix of predicting sarcopenia of the monomodal model based on ultrasound images. (C) The confusion matrix of predicting sarcopenia of the bimodal model based on ultrasound images and clinical information. (D) The confusion matrix of predicting sarcopenia of the bimodal model based on ultrasound images and laboratory information. (E) The confusion matrix of predicting sarcopenia of the multimodal model based on ultrasound images, clinical information, and laboratory information. (F) The contributions of ultrasound images, clinical information, and laboratory information to the multimodal model’s decision-making. AUC: area under the receiver operator characteristic curve; ROC: receiver operating characteristic.

Based on the ConvNeXt, two bimodal models (ultrasound images and clinical information, and ultrasound images and laboratory information) and one multimodal model (ultrasound images, clinical information, and laboratory information) were developed. The diagnostic performance of the monomodal, bimodal, and multimodal models are shown in Table 2. The multimodal CNN model demonstrated the best diagnostic performance statistically, with an AUC of 0.845 and an *F*_1_-score of 0.754 (Figure 4A), showing differences compared to the best monomodal model (AUC: 0.845 vs 0.827; *P*=.02), as well as the two bimodal models based on ultrasound images combined with clinical information (AUC: 0.845 vs 0.826; *P*=.03) and with laboratory information (AUC: 0.845 vs 0.832; *P*=.04). The confusion matrices showed that the multimodal model performed the highest level of correct prediction of sarcopenia (77), followed by the monomodal model based on only ultrasound images (74; Figure 4B-E).

**Table 2:** The diagnostic performance of the monomodal, bimodal, and multimodal models.

Ratings	Ultrasound images	Ultrasound images + clinical information	Ultrasound images + laboratory information	Ultrasound images + clinical information + laboratory information
Sensitivity	0.733	0.689	0.722	0.754
Specificity	0.782	0.791	0.794	0.799
Accuracy	0.770	0.761	0.773	0.788
AUC^a^	0.827	0.826	0.832	0.845
*F*_1_-score	0.738	0.721	0.737	0.754

^a^AUC: area under the receiver operator characteristic curve.

The SHAP values were additionally analyzed to understand the relative contributions of each data type to the overall classification results (Figure 4F). In the multimodal model, the ultrasound images contributed the highest percentage to diagnosing sarcopenia (0.787) and nonsarcopenia (0.823).

### Sensitivity Analysis

The sensitivity analysis was conducted to evaluate the potential impact of imputing missing values. A total of 86 cases with missing data were excluded, and model analysis was performed on the remaining 271 cases with complete data. The number of cases with missing features is summarized in Multimedia Appendix 7. The diagnostic performance of three monomodal models based on complete data is shown in Table 3. The clinical information model achieved an AUC of 0.787 and an *F*_1_-score of 0.630, and the laboratory information model achieved an AUC of 0.666 and an *F*_1_-score of 0.480. The ultrasound images model achieved the highest AUC of 0.851 and *F*_1_-score of 0.698. The diagnostic performance of the monomodal, bimodal, and multimodal models based on complete data is shown in Table 4. Although the multimodal CNN model achieved the best diagnostic performance statistically (AUC=0.883; *F*_1_-score=0.728), SHAP analysis revealed that ultrasound images still contributed the most to diagnosing sarcopenia (0.810) and nonsarcopenia (0.795). Finally, we selected the monomodal model based on only ultrasound images as the optimal one for diagnosing sarcopenia and named it the SARCO model.

**Table 3:** The diagnostic performance of three monomodal models based on complete data.

Ratings	Ultrasound images	Clinical information	Laboratory information
Sensitivity	0.808	0.712	0.506
Specificity	0.793	0.776	0.754
Accuracy	0.797	0.758	0.682
AUC^a^	0.851	0.787	0.666
*F*_1_-score	0.698	0.630	0.480

^a^AUC: area under the receiver operator characteristic curve.

**Table 4:** The diagnostic performance of the monomodal, bimodal, and multimodal models based on complete data.

Ratings	Ultrasound images	Ultrasound images + clinical information	Ultrasound images + laboratory information	Ultrasound images + clinical information + laboratory information
Sensitivity	0.808	0.739	0.739	0.808
Specificity	0.793	0.821	0.798	0.832
Accuracy	0.797	0.797	0.781	0.825
AUC^a^	0.851	0.866	0.854	0.883
*F*_1_-score	0.698	0.679	0.662	0.728

^a^AUC: area under the receiver operator characteristic curve.

### External Validation and Proof-of-Concept Validation of the SARCO Model

**Figure 5:**
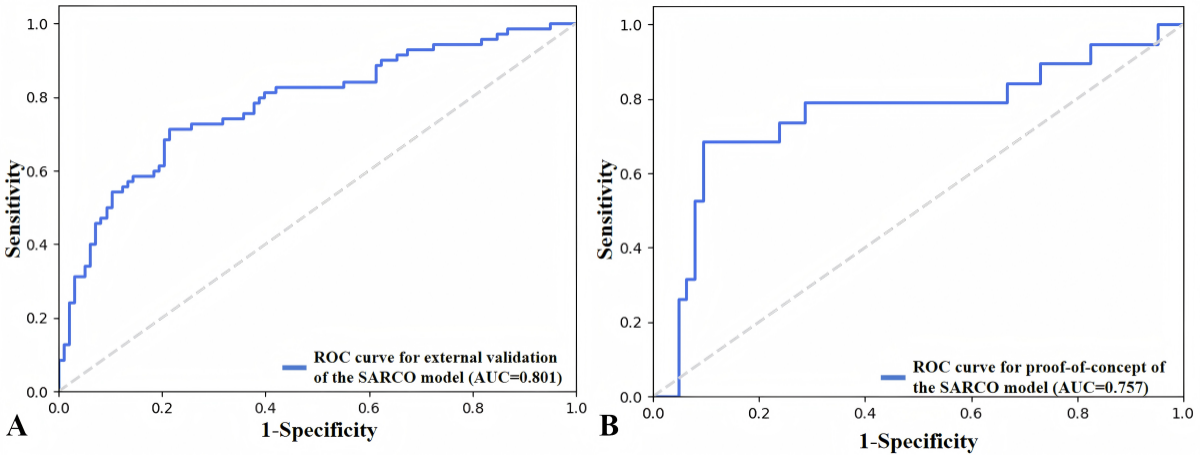
The diagnostic performance of the SARCO model. (A) The ROC curve of the SARCO model in the external validation. (B) The ROC curve of the SARCO model in the proof-of-concept validation. AUC: area under the receiver operator characteristic curve; ROC: receiver operating characteristic.

The SARCO model achieved excellent prediction performance in external validation, with sensitivity=0.705, specificity=0.794, accuracy=0.753, AUC=0.801, and *F*_1_-score=0.727 (Figure 5A). The SARCO model also achieved satisfactory prediction performance in proof-of-concept validation, with sensitivity=0.789, specificity=0.825, accuracy=0.817, AUC=0.757, and *F*_1_-score=0.666 (Figure 5B).

### Visual Interpretation of the SARCO Model

**Figure 6:**
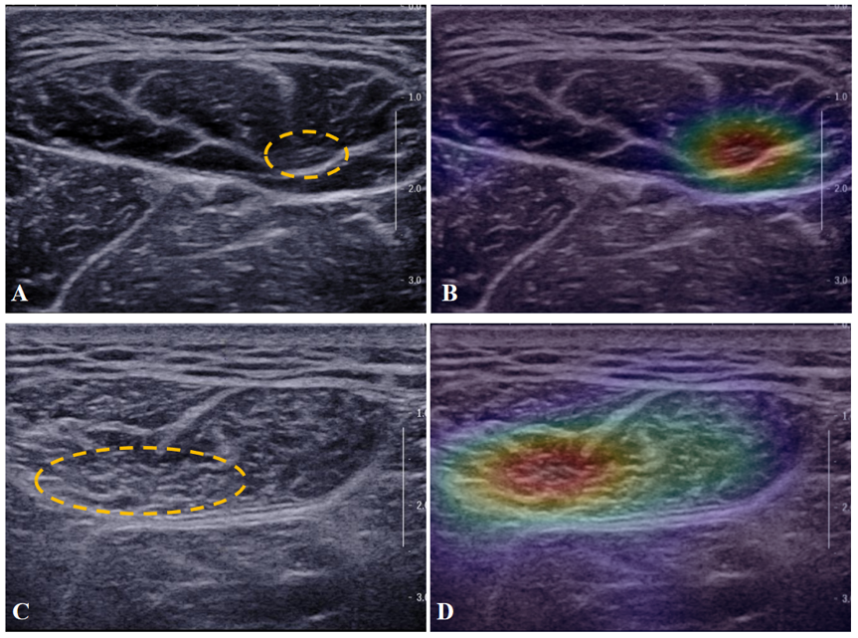
The corresponding heat maps of ultrasound images of the SARCO model. (A and B) The corresponding heat maps of ultrasound images of a participant without sarcopenia. (C and D) The corresponding heat maps of ultrasound images of a participant with sarcopenia. High activation regions are marked with dotted lines.

The corresponding heat maps of the ultrasound images of the SARCO model are presented in Figure 6. The different color distributions in the heat maps indicate the models’ emphasis on the most predictive regions and image features in the context of sarcopenia. The red parts of the heat map indicate that those parts provide more informant features during the network’s predictive process. In contrast to nonsarcopenia (Figure 6A and B), the hyperechoic areas due to muscle fascia or fibrosis involvement are characterized by high activation (Figure 6C and D).

## Discussion

### Principal Findings

In this study, we successfully developed several models using ultrasound images, clinical information, and laboratory information to diagnose sarcopenia. As expected, our findings showed that the multimodal model statistically demonstrated the highest diagnostic performance. Additionally, we noted that ultrasound images played a dominant role compared to clinical and laboratory information in the diagnostic decision-making process. Accordingly, the absolute difference in performance between the monomodal model based on ultrasound images and the multimodal model was minimal. Therefore, considering the accessibility of ultrasound examination, the monomodal model based on ultrasound images was considered the most practiced solution for screening sarcopenia, despite it resulting in some accuracy losses.

The pathological changes of sarcopenia are the foundation for the macroscopic changes in muscle [7,19]. Macroscopic changes encompass alterations in muscle thickness, cross-sectional area, and hardness, all of which can be further reflected in the ultrasound images [19,20]. Muscle thickness and cross-sectional area not only reflect muscle mass but also show a positive correlation with muscle strength and physical function, facilitating the diagnosis of sarcopenia [21,22]. For example, Chen et al [23] developed a diagnostic model for sarcopenia based on age, cross-sectional area changes, and shear wave elasticity value changes, yielding an AUC of 0.883. Tang et al [24] developed an ultrasound-derived muscle assessment system based on muscle thickness, handgrip strength, and gait speed. The system had an overall diagnostic sensitivity of 92.7% and a specificity of 91% for sarcopenia. These studies have proved the effectiveness of conventional ultrasound methods; however, they are time-consuming and performed in diverse protocols. Moreover, it is challenging to detect minor muscle texture abnormalities in the early phase of sarcopenia through apparent macroscopic muscle morphology by conventional imaging methods.

The minor abnormalities in muscle may be identified using advanced image processing and artificial intelligence (AI) techniques. Early screening of individuals with suspected muscle texture abnormalities enables timely referral to hospitals for further evaluation and treatment, thereby optimizing the diagnostic procedure. Recently, some efforts have been made to use AI methods in ultrasound images for sarcopenia assessment. For example, Yang et al [25] demonstrated the feasibility of evaluating muscle mass through quantitative muscle texture features using a gray-level co-occurrence matrix. Wilkinson et al [26] discovered that gray-level co-occurrence matrix parameters extracted from ultrasound images were correlated with muscle function in patients having chronic kidney disease. These methods involving manual or semiautomatic feature extraction of muscle texture features could introduce subjectivity and variability, limiting their generalizability across different datasets [27,28]. CNNs happen to be well suited to address these shortcomings [29,30]. CNNs can automatically handle images from large datasets and various imaging conditions, enhancing their suitability for sarcopenia screening.

Exploration of CNN models for diagnosing sarcopenia based on ultrasound images is currently insufficient. Yi et al [31] used three CNNs (VGG19, ResNet50, and DenseNet121) for predicting sarcopenia, achieving accuracies of 0.65-0.75 (based on grayscale ultrasound images) and 0.70-0.80 (based on shear-wave elastography images). Their study primarily focused on assessing the applicability of different CNNs for predicting sarcopenia based on ultrasound images. In this study, we compared three networks and selected the superior-performing ConvNeXt. ConvNeXt, inspired by ResNeXt, improves feature expression through group convolutions, enhancing network depth and width. It allows for better extracting rich structural and detailed information in ultrasound images of sarcopenia [32].

Compared to other studies, another methodological advantage of this study is integrating clinical and laboratory information with ultrasound images to improve the performance of predicting sarcopenia. As expected, the complementary of different data types typically synergistically enhances disease detection. However, based on our results, the improvement in the performance of multimodal models was slight. Clinical and laboratory information indirectly reflects the pathophysiological changes of sarcopenia and may be influenced by external factors, and thus, may not have enough sensitivity to diagnose chronic conditions. Additionally, in the real world, it is difficult to obtain complete clinical and laboratory information from bedridden or housebound older individuals.

On the contrary, morphological changes, such as reduced muscle mass and muscle fiber atrophy, are the most direct manifestations of sarcopenia and can be visualized through imaging methods. Moreover, ultrasound devices possess the advantages of accessibility, mobility, radiation-free, and cost-effectiveness compared with CT and dual-energy x-ray absorptiometry, making them preferable for community preventive services or chronic health care. Though the performance of the SARCO model in a small-scale, proof-of-concept validation was not excellent enough, considering the participants with decompensated cirrhosis were all in weak, bedridden, or nearly disabled conditions, this study preliminarily confirmed the benefits of the SARCO model, which provides theoretical and practical support for large-scale data verification in the future.

This study represented an initial exploration of the use of ultrasound images to diagnose sarcopenia and an important step toward clinical application. However, there were still some limitations, and the model’s accuracy has room for further improvement. First, the sample size for model training and validation was relatively small, although this study applied cross-validation to mitigate biases and errors caused by the sample distribution. Second, our data were obtained from Chinese single-race cohorts. Third, in order to reduce the influence of other tissues on the feature extraction, we use manual segmentation to separate muscle regions. Fourth, some cases lacked partial clinical or laboratory data. Although our sensitivity analysis demonstrated consistent performance trends, future work should address this issue by collecting a more comprehensive dataset and exploring advanced imputation techniques, such as multiple imputation or machine learning-based methods. Finally, it was better to evaluate our SARCO model on public datasets. Unfortunately, we did not find any database containing muscle ultrasound images of sarcopenia.

With an aging population, sarcopenia diagnosis models hold significant potential for clinical application. Moving forward, we will focus on improving the model’s accuracy and clinical applicability through several steps. First, enhancing data quality, optimizing algorithms, optimizing image processing techniques, and developing automatic segmentation techniques will help improve the model’s accuracy and reliability. Second, incorporating more imaging and clinical data, such as CT data and laboratory biomarkers, will enhance the model’s multidimensional predictive capabilities. Third, expanding the sample size to include diverse populations in terms of age, gender, ethnicity, and health status will ensure broader applicability and generalizability. Fourth, external validation across multiple centers and regions is necessary to assess the model’s performance in different populations and improve its clinical adaptability. Finally, beyond the CNNs applied in this study, advanced AI techniques hold promise for improving sarcopenia assessments. For instance, self-supervised learning can mitigate the limitations of labeled data by leveraging large-scale unlabeled ultrasound images for pretraining, enhancing model generalizability. Federated learning enables multicenter collaboration without compromising data privacy, promoting robust and diverse model training [33]. Additionally, the combination of medical imaging with large language models for contextual understanding and predictive analysis could further enhance personalized sarcopenia risk assessment [34]. These advancements, combined with ongoing improvements in data quality and standardization, and the collaboration of physicians and engineers, have the potential to enhance the accuracy and clinical utility of sarcopenia assessments.

### Conclusions

In conclusion, ultrasound images, clinical information, and laboratory information all contributed to diagnosing sarcopenia; however, ultrasound images played a dominant role in model decision-making. Both external and proof-of-concept evaluations have demonstrated that the SARCO model can be a feasible and convenient solution for diagnosing sarcopenia. This advancement holds promise for large-scale sarcopenia screening and health care management of older individuals in clinical or community settings.

## Appendix

Multimedia Appendix 1 The flow diagram of the inclusion and exclusion.

Multimedia Appendix 2 The ultrasound equipment and transducer of the centers.

Multimedia Appendix 3 Schematic diagram of muscle segmentation on ultrasound images.

Multimedia Appendix 4 Development and architecture of three CNN models based on ultrasound images. CNN: convolutional neural network.

Multimedia Appendix 5 The diagnostic performance of the 5-fold cross-validation of three networks based on only ultrasound images.

Multimedia Appendix 6 The comparison of the AUCs and the F1-score of three networks based on only ultrasound images. AUC: area under the receiver operator characteristic curve.

Multimedia Appendix 7 Pie chart of cases with missing clinical and laboratory features.

## Abbreviations

AI: artificial intelligence
AUC: area under the receiver operator characteristic curve
CNN: convolutional neural network
CONSORT-EHEALTH: Consolidated Standards of Reporting Trials of Electronic and Mobile Health Applications and Online Telehealth
CT: computed tomography
GELU: Gaussian error linear unit
MLP: multilayer perceptron
RF: rectus femoris
ROC: receiver operating characteristic
SHAP: Shapley additive explanation

## Data Availability

The datasets generated or analyzed during this study are available from the corresponding author upon reasonable request.
