# Supplementary material for: Diagnosis of Sarcopenia Using Convolutional Neural Network Models Based on Muscle Ultrasound Images: Prospective Multicenter Study
Source: J Med Internet Res. 2025 May 6;27:e70545. doi: 10.2196/70545 (PMC12057287; doi:10.2196/70545)
Supplement: Multimedia Appendix 1 [file jmir_v27i1e70545_app1.docx]

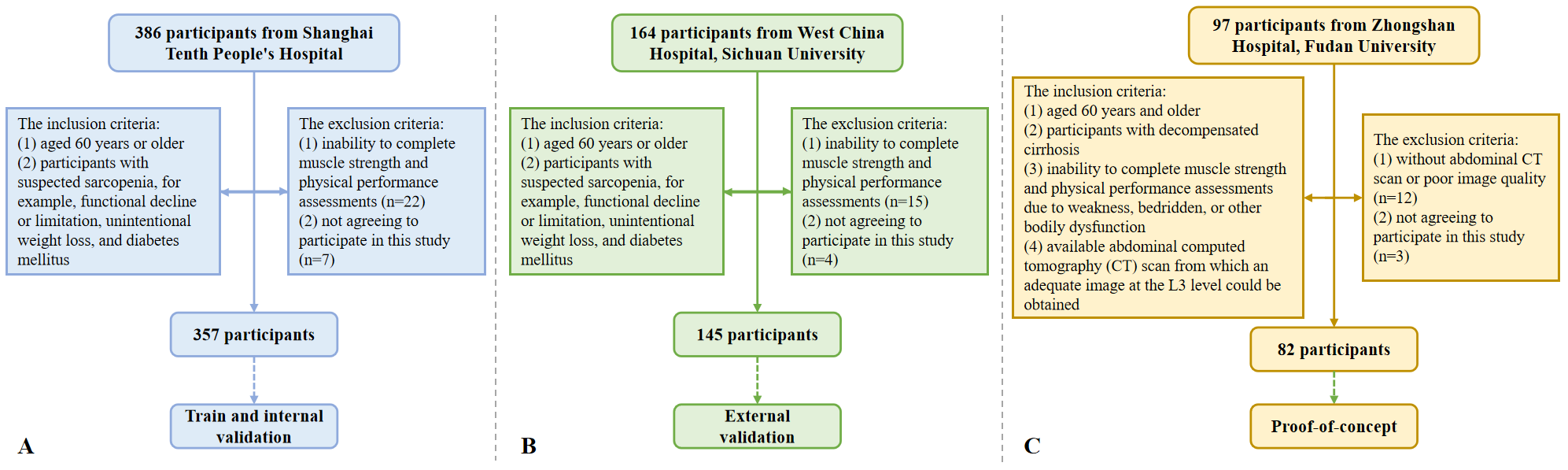
**A.** The flow of inclusion and exclusion of training and internal validation group; **B.** The flow of inclusion and exclusion of external validation group; and **C.** The flow of inclusion and exclusion of proof-of-concept group.
