# Supplementary material for: Diagnosis of Sarcopenia Using Convolutional Neural Network Models Based on Muscle Ultrasound Images: Prospective Multicenter Study
Source: J Med Internet Res. 2025 May 6;27:e70545. doi: 10.2196/70545 (PMC12057287; doi:10.2196/70545)
Supplement: Multimedia Appendix 2 [file jmir_v27i1e70545_app2.docx]

| Cohort | Center | US equipment | US transducer |
| --- | --- | --- | --- |
| Training and internal validation cohort | Shanghai Tenth People's Hospital | Aixplorer Ultrasound system (SuperSonic Imagine, Aix-en-Provence, France) | SL 10–2 multifrequency linear transducer |
| External validation cohort | West China Hospital, Sichuan University | Aixplorer Ultrasound system (SuperSonic Imagine, Aix-en-Provence, France) | SL 10–2 multifrequency linear transducer |
|  |  | Mindray M9 Portable Ultrasound Machine (Mindray, Shenzhen, China) | SL 10–3 multifrequency linear transducer |
|  |  | Mindray MX7 Portable Ultrasound Machine (Mindray, Shenzhen, China) | SL 13–3 multifrequency linear transducer |
| Proof-of-concept cohort | Zhongshan Hospital, Fudan University | R10 Prestige ultrasound system (Samsung Medison Co. Ltd., Hongcheon, Korea) | SL 12–3 multifrequency linear transducer |
