# Supplementary material for: Diagnosis of Sarcopenia Using Convolutional Neural Network Models Based on Muscle Ultrasound Images: Prospective Multicenter Study
Source: J Med Internet Res. 2025 May 6;27:e70545. doi: 10.2196/70545 (PMC12057287; doi:10.2196/70545)
Supplement: Multimedia Appendix 3 [file jmir_v27i1e70545_app3.docx]

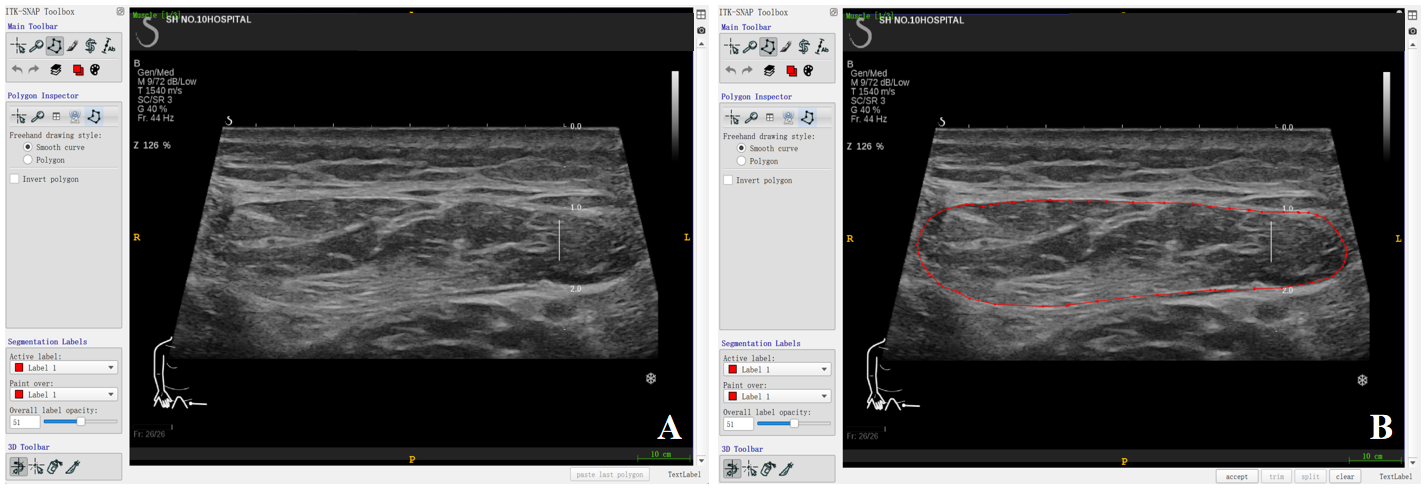


1. US image of muscle without segmentation; **B**. Segmenting muscle region (red circle) in US image using ITK-SNAP software.
