# Supplementary material for: Diagnosis of Sarcopenia Using Convolutional Neural Network Models Based on Muscle Ultrasound Images: Prospective Multicenter Study
Source: J Med Internet Res. 2025 May 6;27:e70545. doi: 10.2196/70545 (PMC12057287; doi:10.2196/70545)
Supplement: Multimedia Appendix 4 [file jmir_v27i1e70545_app4.docx]

**EfficientNet:** EfficientNet proposes a compound scaling method, which uses the NAS (neural network architecture search) method to expand the network by combining width, depth, and resolution. The EfficientNet is constructed by stacking multiple MBConv Blocks. MBConv Block consists of two 1x1 convolution layers, a depthwise convolution layer, and an SE layer. One of the 1x1 convolutions is used to reduce the dimension of the feature map, and the other is used to raise the dimension.

**ConvNeXt:** ConvNeXt aims to improve classical CNNs by utilizing transformer design components to refine CNNs further to reach their maximum potential. With a deep study of the macro design, ResNeXt, large kernel size, inverted bottleneck, and various layer-wise micro designs, ConvNeXt emerges as an outstanding feature extractor for image classification.

**Swin Transformer:** Swin Transformer is constructed by stacking multiple Swin Transformer Blocks, which have been inherited from the general Visual Transformer (ViT) architecture. In Swin Transformer Blocks, multiheaded self-attention (MSA) blocks of ViT are replaced with an alternate ordering of window-based MSA (W-MSA) and shifted window-based MSA (SW-MSA). The shift windows bring greater efficiency and significantly reduce the sequence length because self-attention is calculated in the window. At the same time, the interaction between two adjacent windows can be made through the operation of shifting (movement).
