# Supplementary material for: Diagnosis of Sarcopenia Using Convolutional Neural Network Models Based on Muscle Ultrasound Images: Prospective Multicenter Study
Source: J Med Internet Res. 2025 May 6;27:e70545. doi: 10.2196/70545 (PMC12057287; doi:10.2196/70545)
Supplement: Multimedia Appendix 5 [file jmir_v27i1e70545_app5.docx]

| Folds and models | Sensitivity | Specificity | Accuracy | AUC |
| --- | --- | --- | --- | --- |
| Fold1 | | | | |
| EfficientNet | 0.714 | 0.726 | 0.723 | 0.821 |
| Swin Transformer | 0.786 | 0.706 | 0.723 | 0.818 |
| ConvNeXt | 0.714 | 0.804 | 0.785 | 0.825 |
| Fold2 | | | | |
| EfficientNet | 0.750 | 0.659 | 0.692 | 0.760 |
| Swin Transformer | 0.875 | 0.463 | 0.615 | 0.793 |
| ConvNeXt | 0.833 | 0.610 | 0.692 | 0.788 |
| Fold3 | | | | |
| EfficientNet | 0.533 | 0.824 | 0.758 | 0.761 |
| Swin Transformer | 0.667 | 0.882 | 0.833 | 0.821 |
| ConvNeXt | 0.733 | 0.824 | 0.803 | 0.864 |
| Fold4 | | | | |
| EfficientNet | 0.889 | 0.702 | 0.754 | 0.832 |
| Swin Transformer | 0.778 | 0.787 | 0.785 | 0.859 |
| ConvNeXt | 0.778 | 0.745 | 0.754 | 0.835 |
| Fold5 | | | | |
| EfficientNet | 0.609 | 0.814 | 0.742 | 0.838 |
| Swin Transformer | 0.565 | 0.907 | 0.788 | 0.888 |
| ConvNeXt | 0.609 | 0.930 | 0.818 | 0.826 |
| Mean of 5 folds | | | | |
| EfficientNet | 0.699 | 0.745 | 0.734 | 0.802 |
| Swin Transformer | 0.734 | 0.749 | 0.749 | 0.836 |
| ConvNeXt | 0.733 | 0.782 | 0.770 | 0.827 |
