# Supplementary figures and images for: Diagnosis of Sarcopenia Using Convolutional Neural Network Models Based on Muscle Ultrasound Images: Prospective Multicenter Study
Source: J Med Internet Res. 2025 May 6;27:e70545. doi: 10.2196/70545 (PMC12057287; doi:10.2196/70545)

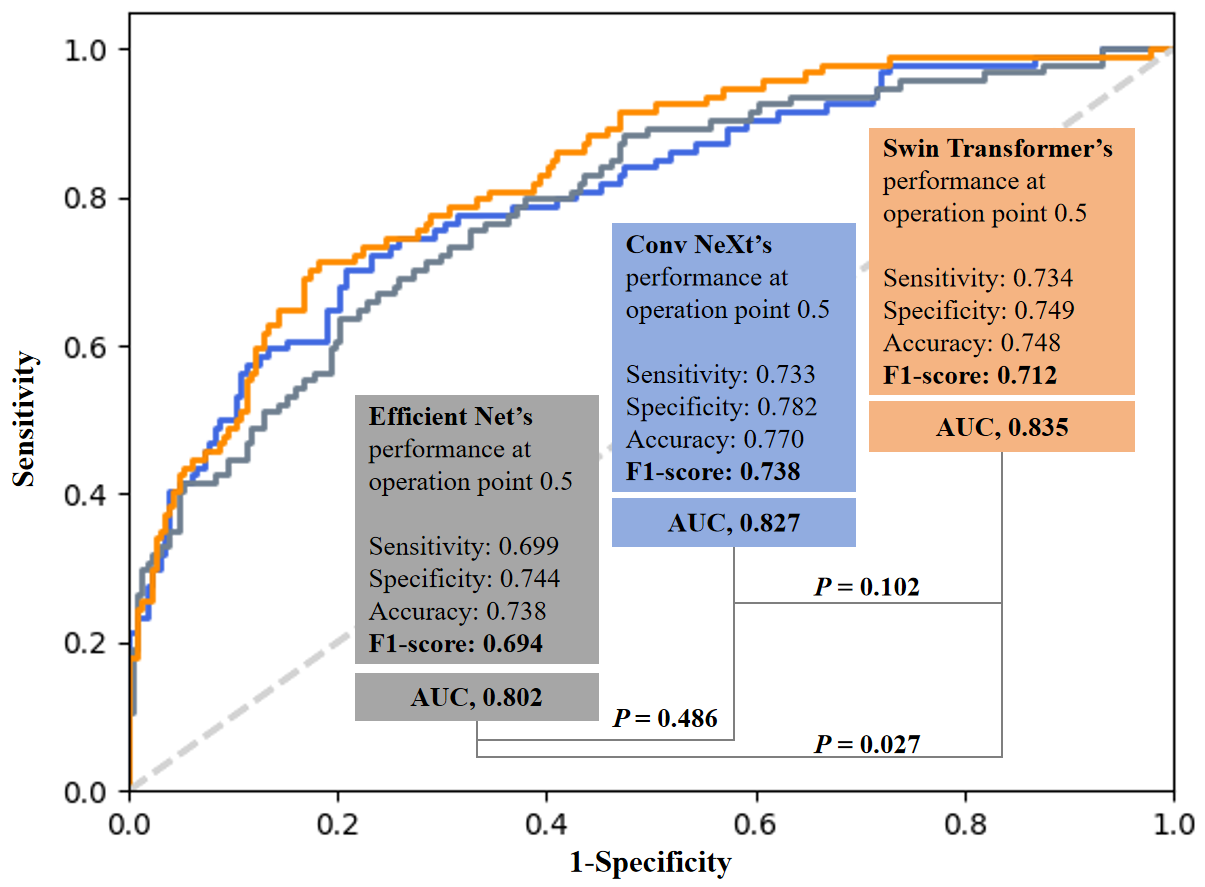

Supplement: Multimedia Appendix 6 [file jmir_v27i1e70545_app6.docx]
