# Supplementary material for: Diagnosis of Sarcopenia Using Convolutional Neural Network Models Based on Muscle Ultrasound Images: Prospective Multicenter Study
Source: J Med Internet Res. 2025 May 6;27:e70545. doi: 10.2196/70545 (PMC12057287; doi:10.2196/70545)
Supplement: Multimedia Appendix 7 [file jmir_v27i1e70545_app7.docx]

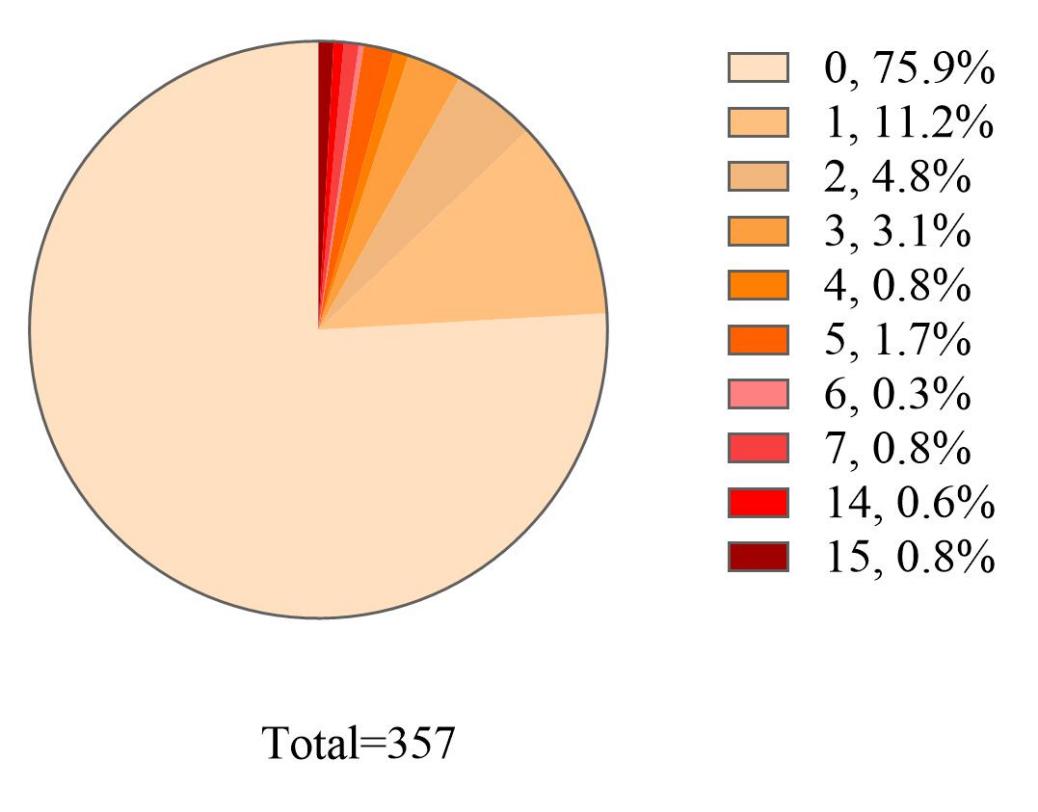
The legend indicates the number of missing features and the corresponding percentage of cases relative to the total number of cases. This study included a total of 357 cases. Among all cases, 271 (75.9%) cases had complete data. 40 (11.2%) cases had one missing feature, 17 (4.8%) cases had two missing features, 11 (3.1%) cases had three missing features, 3 (0.8%) cases had four missing features, 6 (1.7%) cases had five missing features, 1 (0.3%) case had six missing features, 3 (0.8%) cases had seven missing features, 2 (0.6%) cases had fourteen missing features, and 3 (0.8%) cases had fifteen missing features.
